# AGE FRIENDLINESS OF COMMUNITIES CONTRIBUTES TO QUALITY OF LIFE

**DOI:** 10.1093/geroni/igz038.2087

**Published:** 2019-11-08

**Authors:** Charles Seguin, Nadia Mullen, Arne Stinchcombe, Shawn Marshall, Gary Naglie, Mark Rapoport, Bruce Weaver, Michel Bédard

**Affiliations:** 1 Lakehead University, Thunder Bay, Ontario, Canada; 2 Saint Paul University (Ottawa), Ottawa, Ontario, Canada; 3 University of Ottawa/ Ottawa Hospital, Ottawa, Ontario, Canada; 4 Baycrest Health Sciences/University of Toronto, Toronto, Ontario, Canada; 5 Sunnybrook Health Sciences/University of Toronto, Toronto, Ontario, Canada

## Abstract

The World Health Organization (WHO) emphasized the importance of age-friendly communities in supporting quality of life for older adults. We aimed to determine the contribution of the age-friendliness of communities to quality of life in a sample of healthy older adults. We used data collected through a longitudinal study on drivers and ex-drivers. We used the World Health Organization Quality of Life instrument (WHOQOL-BREF; WHOQOL Group, 1998) to measure physical health, psychological health, social relationships, and environment. We used the Age-Friendly Survey (AFS; Menec & Nowicki, 2014) to measure 9 domains of participants’ perceptions of community age-friendliness. We estimated 4 multivariable linear regression models. The dependent variables were the 4 domains of the WHOQOL-BREF. Each model had AFS as the focal independent variable and participants’ age, gender, health status, and depression symptoms as control variables. Data from 171 participants were available; mean age was 83.2 years (SD=4.1), 61% were women. Most participants reported a good health status and few depression symptoms. The models explained between 18 and 27% of the variance in WHOQOL scores; community age-friendliness was a statistically significant variable in all models, accounting for 2-3% of the variance. The identification of factors that contribute to quality of life will serve as the foundation upon which policies and interventions to promote successful and healthy aging can be developed. Future work will require consideration of the specific aspects of communities that may affect quality of life the most and that have the most potential for modification.

